# A phase II basket trial of vosoritide in children with RASopathies, ACAN, and NPR2 deficiency

**DOI:** 10.1210/clinem/dgag157

**Published:** 2026-04-10

**Authors:** Andrew Dauber, Anqing Zhang, Niusha Shafaei, Raheem Seaforth, Kimberly Pitner, Niti Dham, Roopa Kanakatti Shankar, Nadia Merchant

**Affiliations:** Division of Endocrinology, Children's National Hospital, Washington, DC 20010, USA; Department of Pediatrics, The George Washington University School of Medicine and Health Sciences, Washington, DC 20052, USA; Division of Biostatistics, Children's National Hospital, Washington, DC 20010, USA; Division of Endocrinology, Children's National Hospital, Washington, DC 20010, USA; Division of Endocrinology, Children's National Hospital, Washington, DC 20010, USA; Division of Endocrinology, Children's National Hospital, Washington, DC 20010, USA; Department of Pediatrics, The George Washington University School of Medicine and Health Sciences, Washington, DC 20052, USA; Division of Cardiology, Children's National Hospital, Washington, DC 20010, USA; Division of Endocrinology, Children's National Hospital, Washington, DC 20010, USA; Department of Pediatrics, The George Washington University School of Medicine and Health Sciences, Washington, DC 20052, USA; Division of Pediatric Endocrinology, Department of Pediatrics, University of Texas Southwestern Medical Center, Dallas, TX 75390, USA

**Keywords:** vosoritide, aggrecan, NPR2, Noonan syndrome, RASopathy, C-type natriuretic peptide

## Abstract

**Context:**

Genetic defects in many biological pathways, including activation of the Ras-MAPK pathway, cause short stature. Vosoritide, a C-type natriuretic peptide analog that inhibits this pathway, has been approved for use in achondroplasia.

**Objective:**

To determine whether vosoritide improves growth in children with disorders of the Ras-MAPK pathway, including RASopathies, ACAN, and NPR2 deficiency.

**Design:**

Prospective Phase 2 basket trial

**Setting:**

Academic medical center

**Participants:**

Thirty prepubertal children aged 3 to 11 years with a RASopathy, ACAN or NPR2 deficiency and height ≤ −2.25 SD

**Intervention:**

Six-month observation period followed by 12-month treatment with vosoritide subcutaneously 15 µg/kg/day.

**Main Outcome Measures:**

Co-primary outcomes included incidence of adverse events, change in annualized growth velocity (AGV), and height standard deviation scores.

**Results:**

The AGV increased from 4.53 ± 1.61 cm/year to 8.09 ± 1.58 cm/year with treatment (*P* < .0001). This corresponded to a 4.0 SD (95%CI 3.08-4.91) increase in age and sex-adjusted AGV Z-score (*P* < .0001). The increase in AGV was seen in all genetic subgroups. There was a height increase of 0.65 SD (95%CI 0.53-0.77) in the treatment vs observation period (*P* < .0001).

Short-term safety was reassuring, with mild injection site reactions being the most common adverse events. However, with longer use, 5 subjects discontinued medication due to adverse events, including 3 slipped capital femoral epiphyses and 4 cases of genu valgum.

**Conclusion:**

Vosoritide led to marked increases in growth velocity in children with RASopathies, ACAN, and NPR2 deficiency, raising the possibility that vosoritide could be an effective precision medicine for all growth disorders affecting the MAPK pathway.

Hundreds of biological pathways contribute to normal growth throughout childhood, and many of these pathways influence linear growth via modulation of chondrocyte biology in the growth plate. Genetic defects affecting these pathways can lead to short stature, including syndromic forms and primary skeletal dysplasia. Despite much being known about the biology of growth, until recently, recombinant human growth hormone was essentially the only medication available to promote growth in children with short stature. In 2021, vosoritide, a C-type natriuretic peptide analog, was approved for the treatment of short stature in children with achondroplasia. Vosoritide promotes growth through inhibition of the Ras-MAPK (mitogen-activated protein kinase) pathway. This pathway is downstream of FGFR3, the protein in which activating variants lead to achondroplasia and hypochondroplasia. We conducted a Phase 2 basket trial of vosoritide in patients with rare, genetic causes of short stature, which also lead to activation of the Ras-MAPK pathway. We previously reported the results of the trial for the cohort of children with hypochondroplasia (1). Herein, we report the results of all other subjects in the basket trial, including the RASopathy, ACAN, and NPR2 deficiency cohorts.

RASopathies are a group of syndromes marked by dysregulation of genes in the germline Ras-MAPK pathway, increasing downstream inhibitory signaling on the growth plate. Clinical manifestations include short stature, dysmorphic features, skeletal dysplasia, cardiovascular, skin, and neurological issues, including developmental delays. Noonan syndrome is the most common RASopathy and ∼40% to 70% of individuals with Noonan syndrome will have short stature (2, 3). Recombinant human growth hormone is approved for use in children with Noonan syndrome and short stature, but the efficacy is quite variable and wanes over time. *NPR2* encodes the receptor for C-type natriuretic peptide. Biallelic pathogenic variants in *NPR2* lead to the severe skeletal dysplasia, Acromesomelic Dysplasia Maroteaux Type, characterized by severe short stature and brachydactyly. Heterozygous variants in *NPR2* have been shown to be the etiology of approximately 1% to 2% of cases of children presenting with idiopathic short stature (4). *ACAN* encodes aggrecan, a proteoglycan found in the extracellular matrix of cartilage in the growth plates and on articular surfaces. Individuals with heterozygous mutations in ACAN present with short stature, often with advanced bone age and premature growth cessation (5). Similar to *NPR2*, *ACAN* variants are one of the most common genetic etiologies of idiopathic short stature (4). In a chick model of aggrecan deficiency, increased levels of activated MAPK were seen in growth plate cartilage (6). There are no currently approved treatments for either NPR2 or ACAN deficiency.

## Materials and methods

### Study design

The full methods of the study have been published previously (1), and the full protocol is available in the supplementary data (7). In brief, this is a Phase 2, open-label basket trial of vosoritide in children with specific, rare genetic causes of short stature conducted at a single site, Children's National Hospital, in Washington, DC (Study Pro00013585, ClinicalTrials.gov number NCT04219007). The study consists of a 6-month observation period followed by a 12-month treatment period. Vosoritide was administered daily via subcutaneous injection at a dose of 15 µg/kg/day.

Eligible participants included prepubertal children between the ages of 3 and 11 years for males and 3 and 10 years for females with 1 of 6 rare, genetic causes of short stature, including: 1. Hypochondroplasia, 2. RASopathies, 3. *ACAN* pathogenic/likely pathogenic variant carriers, 4. Carriers of heterozygous pathogenic/likely pathogenic variants in *NPR2*, 5. SHOX deficiency, and 6. Carriers of pathogenic/likely pathogenic variants in the *NPPC* gene (the gene that encodes C-type natriuretic peptide). Subjects were required to have a height < −2.25 SDS, the currently FDA approved threshold for growth hormone therapy for idiopathic short stature. Full inclusion and exclusion criteria are listed in the protocol.

Results for the children with hypochondroplasia were published previously (1). The current manuscript reports the results of all other genetic categories. Study enrollment for all cohorts occurred between August 2020 and February 2024. The final subject completed the 18-month study in August 2025.

The study received ethical approval from the Institutional Review Board of Children's National Hospital. Written informed consent was obtained from each participant's parent or legal guardian. Verbal assents were obtained for all children aged 7 years or older, and written assents were obtained once children turned 12 years old.

### Study outcomes

The study has 3 prespecified co-primary outcomes: 1. Medication safety defined as the rates of adverse events (AEs), 2. Change from baseline in age-sex standardized annualized growth velocity (AGV), and 3. Change from baseline in age-sex standardized height SDS. Secondary outcomes included the change in body proportions as measured by the sitting height to standing height ratio and arm span minus standing height, as well as the change in bone age to chronological age ratio after 12 months of therapy. Bone age X-rays were read by a single reviewer using the Greulich and Pyle methodology, who was blinded to participant ID, diagnosis, age and time point. Exploratory endpoints included collagen X biomarker (CXM) levels, a marker of endochondral ossification and a proxy for growth velocity, and quality of life, assessed using the QoLISSY (Quality of Life in Short Stature Youth) survey (8).

### Statistical analysis

Two subjects discontinued vosoritide treatment after 51 and 107 days, the first of whom dropped out of the study. Due to the short period of intervention, these 2 subjects were removed from the efficacy analysis, but all subjects were included in the safety analysis. Medication adherence was calculated using 2 methods: the number of returned empty vials divided by the number of days of prescribed therapy as well as through self-reported daily injection diaries. Primary and secondary clinical outcomes were summarized with mean (SD) for the 6-month observation period and the 12-month treatment period. Paired *t*-tests were carried out to compare the difference between the treatment and observation periods. Safety data (AEs) were summarized as count and percentage of patients as well as the number of total occurrences per each listed AE for the observation period and the treatment period, respectively. As an exploratory analysis, the primary endpoints were stratified based on genetic subgroup. Details of the statistical analysis are available in the full statistical analysis plan in the supplementary data (7). Analysis was performed by Ad and AZ.

### Role of the funding source

This is an investigator-initiated study funded by a grant from BioMarin Pharmaceutical. The investigator team designed and wrote the protocol, conducted the study, performed all analyses, and composed the manuscript. BioMarin played no role in any of these functions. An independent data and safety monitoring board provided oversight and reviewed all data every 6 months.

## Results

### Participants

**Figure 1:**
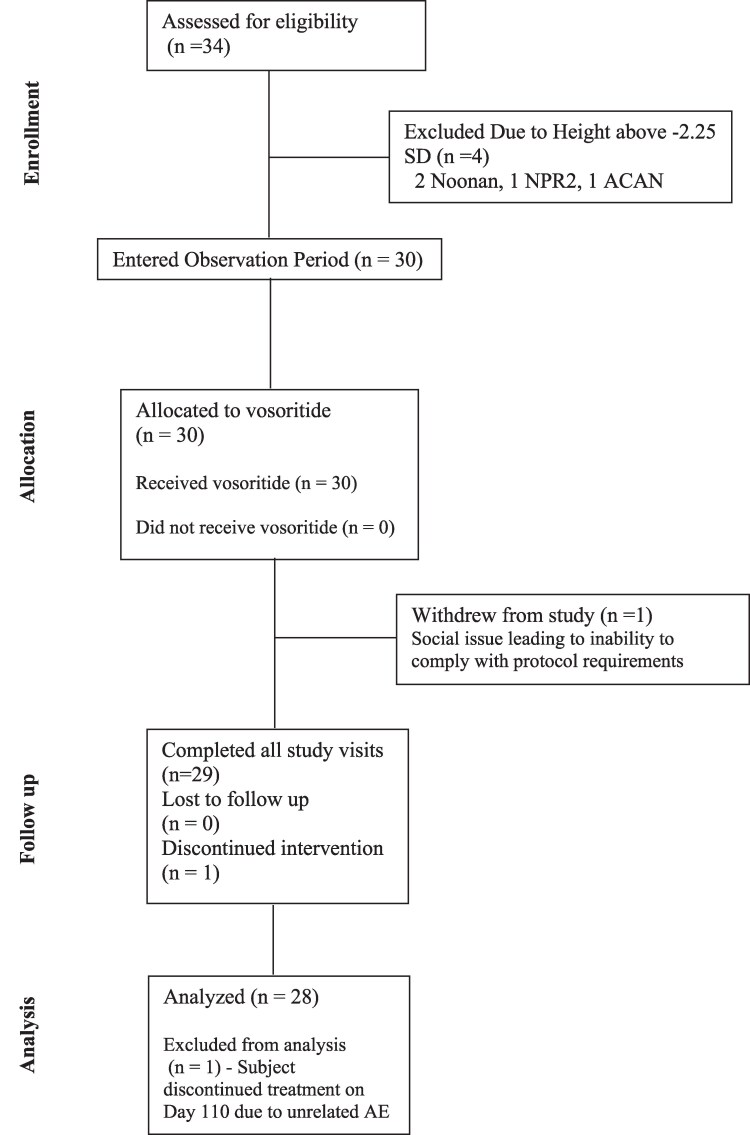
CONSORT diagram.

Thirty participants enrolled in the study and were initiated on vosoritide treatment. One subject withdrew from the study after 51 days of treatment due to social issues, leading to an inability to comply with protocol requirements. A second subject discontinued the vosoritide intervention after 110 days of treatment due to an adverse event unrelated to vosoritide treatment. The remaining 28 participants completed the trial and are included in the efficacy analysis (Fig. 1). Mean (SD) age at screening was 7.2 ± 2.2 years (Table 1). Eight subjects (27%) were female. Twelve (40%) subjects had *ACAN* variants, 7 (23%) had *NPR2* variants, and 11 subjects (37%) had RASopathies, including 8 subjects with Noonan syndrome, 2 with Neurofibromatosis type 1 and 1 with Costello syndrome. Five subjects (17%) had previously been treated with growth hormone. Additional details of individual subject's medical history, genetic variants, and anthropometrics are available in Tables S1 + S2 (7). After excluding the 2 subjects who discontinued treatment, adherence to daily vosoritide administration was excellent, with a median administration rate of 99.3% (range 84.3-100%) based on vial counts and 99.3% (range 91.4-100%) based on daily diaries.

**Table 1:** Demographics and clinical characteristics

Total enrolled subjects	*N* = 30
Age at screening (years)	
Mean (SD); median (IQR)	7.17 (2.22); 7.21 (3.01)
Age group # (%)	
3 to <5 year	6 (20%)
5 to <9 year	18 (60%)
9 to <11 year	6 (20%)
Sex	
Female	8 (26.7%)
Male	22 (73.3%)
Race	
Caucasian	19 (63.3%)
Asian	4 (13.3%)
Other	7 (23.3%)
Ethnicity	
Non-Hispanic/Latino	24 (80%)
Hispanic/Latino	6 (20%)
Previously treated with growth hormone	
Yes	5 (16.7%)
No	25 (83.3%)
Genetics	
NPR2	7 (23.2%)
RASopathy	11 (36.7%)
ACAN	12 (40%)
Height SDS at baseline	
−2.25 to −3	19 (63.3%)
<−3 to −4	10 (33.3%)
<−4	1 (0.3%)

### Height efficacy outcomes (Table 2)

**Figure 2.**
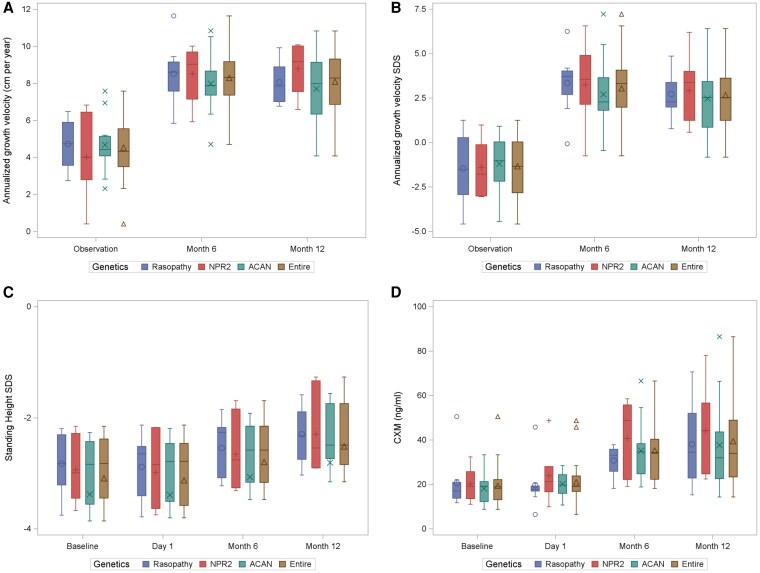
A. Absolute annualized growth velocity. B. Annualized growth velocity Z-score standardized for age and sex. C. Height standard deviation score. D. Collagen X biomarker levels. Note: In Fig. 2C, one subject (VOS22) with a severe form of ACAN deficiency and baseline height of −8.98 SDD was excluded from the Figure for illustrative purposes. Fig. S1 shows the height standard deviation scores while including this subject (7).

The absolute AGV increased from 4.53 ± 1.61 cm/year during the observation period to 8.09 ± 1.58 cm/year during the treatment period for an increase of 3.56 cm/year (*P* < .0001) (Fig. 2A). This corresponded to an increase in age and sex-adjusted AGV Z-score from −1.34 ± 1.64 to 2.66 ± 1.89 SDS, an increase of 4.0 SD (*P* < .0001) (Fig. 2B). Increases in growth velocity were evident at Month 6 and were sustained over the first year. The increase in AGV was seen in all genetic subgroups, with the most prominent increase in absolute AGV seen in the *NPR2* cohort (4.76 cm/year) (Table 2). While all subjects were prepubertal at initiation of vosoritide, 5 subjects (4 males, 1 female) entered puberty during the treatment period. The one female subject had Tanner Stage 2 breasts first noted at her Month 12 visit. Of the 4 male subjects, 2 subjects had 4 cc testes at their 6 Month visits, which progressed to 6 cc at the Month 12 visits, while the other 2 subjects were first noted to have pubertal testes (4-6 cc) at their Month 12 visits.

**Table 2:** Height efficacy outcomes

Primary endpoint	Cohort	Observation period mean (SD)	Treatment period mean (SD)	Difference between treatment and observation (95% CI)	Two-sided *P* value
Annualized growth velocity (cm/year)	All subjects (*N* = 28)	4.53 (1.61)	8.09 (1.58)	3.56 (2.69, 4.43)	<.0001
	NPR2 (*N* = 7)	4.03 (2.19)	8.79 (1.34)	4.76 (2.19, 7.34)	.004
	RASopathy (*N* = 9)	4.72 (1.35)	8.05 (1.10)	3.33 (2.32, 4.35)	<.0001
	ACAN (*N* = 12)	4.68 (1.48)	7.71 (1.96)	3.03 (1.52, 4.54)	.001
Annualized growth velocity *z*-score (SDS)	All subjects (*N* = 28)	−1.34 (1.64)	2.66 (1.89)	4.0 (3.08, 4.91)	<.0001
	NPR2 (*N* = 7)	−1.40 (1.50)	2.91 (1.99)	4.31 (2.50, 6.12)	.001
	RASopathy (*N* = 9)	−1.47 (2.04)	2.73 (1.30)	4.20 (2.59, 5.81)	.0003
	ACAN (*N* = 12)	−1.20 (1.51)	2.47 (2.30)	3.67 (1.85, 5.48)	.001

	Cohort	Baseline mean (SD)	Day 1 mean (SD)	Month 12 Mean (SD)	Change in height SDS during observation period (95% CI)	Change in height SDS during treatment period (95% CI)	Mean difference between treatment and observation (95% CI)	Two-sided *P* value
Standing height SDS	All subjects (*N* = 28)	−3.09 (1.27)	−3.13 (1.33)	−2.52 (1.32)	−0.04 (−0.09, 0.02)	0.61 (0.51, 0.71)	0.65 (0.53, 0.77)	<.0001
	NPR2 (*N* = 7)	−2.94 (0.59)	−2.99 (0.68)	−2.30 (0.72)	−0.05 (−0.19, 0.09)	0.69 (0.49, 0.90)	0.74 (0.46, 1.02)	.0006
	RASopathy (*N* = 9)	−2.83 (0.56)	−2.88 (0.60)	−2.30 (0.56)	−0.06 (−0.19, 0.07)	0.59 (0.41, 0.76)	0.64 (0.51, 0.78)	<.0001
	ACAN (*N* = 12)	−3.38 (1.84)	−3.39 (1.92)	−2.81 (1.89)	−0.01 (−0.11, 0.08)	0.58 (0.37, 0.79)	0.59 (0.33, 0.85)	.0004

Mean baseline height was −3.09 ± 1.27 SD (range −8.99 to −2.13 SD), which did not significantly change during the observation period. At Month 12, mean height increased to −2.52 ± 1.32 SD for a net height increase of 0.65 SD in the treatment vs observation period (*P* < .0001) (Fig. 2C). Again, significant increases were seen in all genetic subgroups, with the greatest increase in the *NPR2* mutation carriers (0.74 SD) (Table 2).

### Safety outcomes (Table 3)

Injection site reactions were common, with 40% of subjects experiencing at least 1 injection site reaction. All injection site reactions were Grades 1 or 2 and resolved without intervention. Rates of AEs occurring in more than 1 subject are shown in Table 3. There were 2 serious adverse events during the treatment period, both of which were deemed unrelated to vosoritide treatment (one episode of status asthmaticus requiring hospitalization and one episode of appendicitis requiring surgery). There was one additional grade 3 AE (a traumatic nose fracture), but no grade 4 or 5 AEs. No subject discontinued treatment during the first year due to a treatment-related adverse event. There were no significant changes in echocardiographic findings and no significant cardiovascular adverse events.

**Table 3:** Adverse events

Patients with any adverse event	Observation period (pretreatment) (# of patients, %; # of occurrences)	Treatment period (Year 1) (# of patients, %; # of occurrences)
Injection-related adverse events		
Injection site erythema	N/A	5 (16.7%; 17)
Injection site pain	N/A	4 (13.3%; 16)
Injection site swelling	N/A	9 (30%; 270)
Injection site urticaria	N/A	6 (20%; 23)
Injection site bruising	N/A	5 (16.7%; 6)
Any Injection site reaction (overall)	N/A	12 (40%; 332)
Other adverse events		
Abdominal pain	3 (10%; 3)	2 (6.7%; 2)
Allergic rhinitis	0	2 (6.7%; 2)
Arthralgia	2 (6.7%; 2)	1 (3.3%; 1)
Conjunctivitis	0	2 (6.7%; 2)
Constipation	0	2 (6.7%; 3)
Cough	5 (16.7%; 5)	6 (20%; 6)
Diarrhea	1 (3.3%; 1)	2 (6.7%; 2)
Ear infection	2 (6.7%; 2)	4 (13.3%; 4)
Eczema	1 (3.3%; 1)	1 (3.3%; 1)
Fatigue	2 (6.7%; 2)	3 (10%; 3)
Fever	3 (10%; 3)	8 (26.7%; 10)
Fracture	3 (10%; 3)	0
Headache	3 (10%; 3)	3 (10%; 3)
Myalgia	2 (6.7%; 2)	4 (13.3%; 4)
Nasopharyngitis	0	3 (10%; 3)
Pain in arms or legs	2 (6.7%; 2)	4 (13.3%; 4)
Rhinorrhea	5 (16.7%; 6)	6 (20%; 8)
Scoliosis	3 (10%; 3)	1 (3.3%; 1)
Sore Throat	1 (3.3%; 1)	1 (3.3%; 1)
Upper respiratory tract infection	1 (3.3%; 1)	10 (33.3%; 11)
Vomiting	2 (6.7%; 2)	8 (26.7%; 14)

Five subjects in the long-term extension phase of the trial discontinued treatment due to treatment-related adverse events. Two subjects, one with Noonan syndrome and one with an *ACAN* mutation, developed severe genu valgum requiring outpatient guided growth surgery. Both subjects were initially continued on vosoritide after discussion with their families and orthopedic surgeons. However, both subjects subsequently developed slipped capital femoral epiphyses requiring surgical intervention and resulting in discontinuation of vosoritide at Days 1078 and 1633 of treatment. A 3rd subject with an *ACAN* mutation also developed a slipped capital femoral epiphysis on Day 493 after previously developing coxa valga. Two additional subjects, both with *ACAN* mutations, developed significant genu valgum, resulting in discontinuation of vosoritide. Additional details of these subjects’ treatment courses, as well as a discussion of all cases of genu valgum, are available in Table S3 (7).

### Secondary outcomes (Table 4)

There was no significant change from Day 1 to Month 12 in bone age to chronological age ratio (0.94 ± 0.20 vs 0.92 ± 0.17, *P* = .22) in the entire cohort nor in any subgroup. Subjects with an *ACAN* variant had a slightly advanced ratio at baseline (1.11 ± 0.17), as expected for this condition, which did not significantly change at month 12 (1.06 ± 0.14) (Table S4) (7).

**Table 4:** Secondary endpoints (*N* = 28)

Secondary endpoints	Baseline mean (SD)	Day 1 mean (SD)	Month 12 mean (SD)	Change during observation period (95% CI)	Change during treatment period (95% CI)	Mean difference between treatment and observation (95% CI)	Two-sided *P* value
Bone age (years)	N/A	7.2 (2.7)	8.0 (2.8)	N/A	0.8 (0.6, 1.1)	N/A	<.0001
Bone age/chronological age	N/A	0.94 (0.20)	0.92 (0.17)	N/A	−0.02 (−0.06, 0.01)	N/A	.22
Sitting height ratio							
Unadjusted	0.56 (0.02)	0.56 (0.02)	0.55 (0.02)	−0.003 (−0.006, 0.001)	−0.008 (−0.01, −0.005)	−0.005 (−0.01, 0.00005)	.05
Age/sex adjusted	2.88 (1.01)	2.95 (0.77)	2.66 (0.92)	0.07 (−0.19, 0.34)	−0.30 (−0.52, −0.08)	−0.37 (−0.79, 0.05)	.08
Arm span minus height							
Unadjusted	−2.61 (3.98)	−2.01 (3.47)	−3.08 (4.23)	0.60 (−0.34, 1.55)	−1.07 (−1.81, −0.33)	−1.67 (−3.0, −0.34)	.02
Age/sex adjusted	−0.49 (1.26)	−0.36 (1.08)	−0.83 (1.28)	0.13 (−0.20, 0.47)	−0.47 (−0.72, −0.23)	−0.61 (−1.07, −0.15)	.01
CXM (ng/mL)	19.37 (8.98)	20.94 (9.17)	39.43 (19.37)	1.76 (−1.57, 5.08)	18.43 (11.45, 25.42)	16.28 (7.25, 25.30)	.001

Note: The observation period is the period between the baseline (screening) visit and Day 1 of treatment. The intervention period is the period between Day 1 and Month 12 of treatment. The mean difference between treatment and observation represents the change during the treatment period minus the change during the observation period.

There was evidence of mild body disproportion at baseline, with subjects having a mean +2.88 SD sitting height to standing height ratio. There was a mild improvement in this ratio during the treatment period, although this was not statistically significant compared to the change in the observation period (*P* = .08). Mean arm span minus height was within the normal range at all time points, although this difference did increase modestly with treatment. Similar trends for body proportions were seen in all genetic etiologies (Table S4) (7). CXM levels, a biomarker of bone elongation, were unchanged during the observation period and rose markedly by Month 6 with sustained increases in all subgroups as Month 12 (Fig. 2D and Table S4) (7). There was a significant improvement in parent-reported quality of life in the physical domain during the intervention period (*P* = .01) (Table S5) (7). There were no other significant changes in quality-of-life scores.

## Discussion

In this Phase 2 basket trial, we evaluated the efficacy of vosoritide in children with RASopathies, ACAN, and NPR2 deficiency, all conditions leading to increased activation of MAPK, resulting in short stature. Vosoritide was effective at increasing annualized growth velocity, with all subgroups having mean gains of >3 cm/year. The cohort with NPR2 deficiency more than doubled their baseline growth velocity, although caution should be taken in interpreting these results due to the small number of subjects in each subgroup. Notably, the increases in growth velocity seen in these cohorts are much larger than those seen in a Phase 3 trial of vosoritide for achondroplasia (1.57 cm/year) (9) or in our previously published results in children with hypochondroplasia (1.81 cm/year) (1). It is possible that vosoritide is more effective in RASopathies, ACAN, and NPR2 deficiency, as its mechanism of action is much more proximate to the molecular pathophysiology than in achondroplasia and hypochondroplasia. FGFR3 signals through multiple pathways, only one of which is targeted by vosoritide. It is plausible that in RASopathies, ACAN, and NPR2 deficiency, increased signaling through MAPK is responsible for a larger proportion of the growth impairment, thus making them more amenable to a precision medicine targeting this pathway. It is notable that the subjects’ parents reported an improvement in the physical domain of quality of life after 1 year of treatment, pointing to the significance of the increase in growth rates. Treatment resulted in a near doubling of CXM levels, a biomarker that has been previously associated with linear growth rates (10). Bone age to chronological age ratio remained stable, suggesting that these short-term height gains may translate into increases in adult height.

Growth hormone is approved for use in children with short stature due to Noonan syndrome, although not for other RASopathies, and has been trialed in small cohorts with ACAN and NPR2 deficiency. Real-world data from a large post-marketing study showed a mean height gain of 0.49 SD in children with Noonan syndrome (11), as compared to 0.59 SD in the current trial. In a trial of growth hormone for ACAN deficiency (12), height increased by 0.62 SD in the first year, very similar to the 0.59 SD seen in the current trial. Similarly, in a real-world study of growth hormone treatment for NPR2 deficiency (13), prepubertal children had a median 0.7 SD increase in height SDS in the first year, almost identical to the 0.69 SD gain in the current study. In all of the aforementioned growth hormone studies, growth rates slowed in the subsequent years. Unlike growth hormone, vosoritide has been reported to lead to sustained increases in growth velocity in children with achondroplasia over multiple years (14). Additional follow-up is needed to determine whether a similar sustained growth rate will be seen in the children in the current study.

While our study focused on growth outcomes, it is important to note that subjects had additional important medical comorbidities, especially the subjects in the RASopathy cohort. Many individuals with RASopathies have underlying congenital heart disease and a subset may develop hypertrophic cardiomyopathy. While some of the subjects in this trial did have congenital heart disease, none of them had significant functional cardiac impairments, and thus, we are unable to comment on the potential effects of vosoritide on cardiac function. There were no concerning echocardiographic changes. Additionally, vosoritide does not substantially cross the blood-brain barrier, so we do not expect any change in neurocognitive function or on the growth of central nervous system neoplasms like the optic pathway gliomas that can be seen in patients with neurofibromatosis type 1. Individuals with aggrecan deficiency can develop early-onset osteoarthritis and osteochondritis dissecans. While it is possible that vosoritide could affect the natural history of these conditions, the children in this study did not have any evidence of these conditions, which usually present in later adolescence or young adulthood, and thus, we are unable to comment on the effect of vosoritide on arthritis risk in this population.

The short-term safety profile was reassuring, with mild injection site reactions being the most common adverse events. However, with longer use, 5 subjects required treatment discontinuation due to musculoskeletal complications, 3 of whom also developed slipped capital femoral epiphyses. Four of the subjects who discontinued treatment had aggrecan deficiency. Genu valgum has rarely been reported as a feature of aggrecan deficiency (15, 16), including a single case report of a patient treated with growth hormone who developed progressive genu valgum requiring surgical correction (16). Thus, it is possible that patients with aggrecan deficiency, and potentially Noonan syndrome, are predisposed to genu valgum, which worsens with rapid growth. Slipped capital femoral epiphyses are known to be a rare adverse effect of growth hormone treatment and occur during periods of rapid growth. However, patients with genetic variants leading to overactivity of NPR2 have been reported to develop slipped capital femoral epiphyses (17). Therefore, it is possible that these skeletal complications are a specific result of vosoritide treatment.

In conclusion, vosoritide led to marked increases in growth velocity in children with RASopathies, ACAN, and NPR2 deficiency, raising the possibility that vosoritide could be an effective precision medicine for all growth disorders affecting the MAPK pathway. Caution should be taken with long-term use, and clinicians should be aware of the potential for the development of genu valgum and slipped capital femoral epiphyses, especially in patients with aggrecan deficiency.

## Data Availability

The de-identified individual participant data that underlie the results reported in this Article (including text, tables, figures, and supplement) will be made available upon request to the corresponding author together with the research protocol, for noncommercial, academic purposes. Requests must include a research proposal clarifying how the data will be used, including proposed analysis methodology. A data use agreement with Children's National Hospital will be required prior to data transfer.

## Clinical trial information

ClinicalTrials.gov NCT04219007
